# Mining the Human Host Metabolome Toward an Improved Understanding of Malaria Transmission

**DOI:** 10.3389/fmicb.2020.00164

**Published:** 2020-02-14

**Authors:** Regina Joice Cordy

**Affiliations:** ^1^Department of Biology, Wake Forest University, Winston-Salem, NC, United States; ^2^Department of Microbiology and Immunology, Wake Forest School of Medicine, Winston-Salem, NC, United States

**Keywords:** plasmodium, malaria, gametocyte, metabolomics, mass spectrometry, transmission, mosquito

## Abstract

The big data movement has led to major advances in our ability to assess vast and complex datasets related to the host and parasite during malaria infection. While host and parasite genomics and transcriptomics are often the focus of many computational efforts in malaria research, metabolomics represents another big data type that has great promise for aiding our understanding of complex host-parasite interactions that lead to the transmission of malaria. Recent analyses of the complement of metabolites present in human blood, skin and breath suggest that host metabolites play a critical role in the transmission cycle of malaria. Volatile compounds released through breath and skin serve as attractants to mosquitoes, with malaria-infected hosts appearing to have unique profiles that further increase host attractiveness. Inside the host, fluctuations in the levels of certain metabolites in blood may trigger increased production of transmission-competent sexual stages (gametocytes), setting the stage for enhanced transmission of malaria from human to mosquito. Together, these recent discoveries suggest that metabolites of human blood, skin and breath play critical roles in malaria transmission. This review discusses recent advances in this area, with a focus on metabolites that have been identified to play a role in malaria transmission and methods that may lead to an improved understanding of malaria transmission.

## Introduction

Technological advancements have been made in recent years in sequencing-based (genomics, transcriptomics, epigenomics) and mass spectrometry-based approaches (proteomics, lipidomics, and metabolomics). These so called ‘omics technologies have enabled researchers to explore various biological contexts at multiple levels, and the integration of these technologies is being used to improve our understanding of a range of human diseases (Hasin et al., 2017). An exciting area in which ‘omics technology has been applied is in the realm of host-microbe interactions, including the complex interplay between microbes and their hosts in both pathogenic (e.g. viral, bacterial, and parasitic) and commensal (e.g. microbiome) relationships. These such interactions between microbes and their human hosts are often quite complex, resulting from co-evolution over massive time scales.

A highly complex interaction exists between *Plasmodium* (the etiological agent of malaria) and its invertebrate and vertebrate hosts, resulting from tens of thousands of years of co-evolution (Loy et al., 2017). In addition to *Plasmodium’s* ability to replicate asexually within the blood cells of vertebrate hosts, *Plasmodium* also undergoes complex stage conversions to successfully transmit itself to the invertebrate host (*Anopheles* mosquito), where it must again undergo a series of stage conversions to be successfully delivered to the next vertebrate host. The propagation of malaria, which is attributed to over 400,000 deaths annually (World Health Organization [WHO], 2018), is therefore highly dependent on the ability of *Plasmodium* to successfully undergo multiple complex stage conversions, and move efficiently between the invertebrate and vertebrates hosts. *Plasmodium* has evolved multiple mechanisms for manipulating its hosts in order to enhance the chances of its successful transmission (Koella, 2005; Cator et al., 2012), and the manipulation of the metabolites present in human blood, skin and breath appears to be one of these such mechanisms.

Metabolomics, the high-throughput profiling of small molecules, has been employed to investigate the role of small molecules (metabolites) in the stage conversions and host exchanges that lead to the successful transmission of malaria. Methods that have been employed to investigate such metabolites run the gamut from functional approaches (e.g. sensory attraction tests), classic metabolic approaches (e.g. fractionation and metabolic labeling), and mass spectrometry-based methods (e.g. gas and liquid chromatography). Within mass-spectrometry-based methods, untargeted big data metabolomics approaches and reference compound-based targeted approaches are both utilized.

In this review, the roles of host metabolites are discussed in the context of malaria transmission, with an emphasis on findings resulting from metabolomics-based investigations. Also discussed are the various ways in which different mass spectrometry-based techniques, computational analyses, and multi-omic modeling approaches can further our knowledge about the impact of host metabolites on malaria transmission.

## Metabolic Windows Into Malaria Transmission

In step with ongoing efforts worldwide to eradicate malaria, there has been an increased focus in recent years on gaining an improved understanding of malaria transmission and in developing novel ways to block and interrupt the cycle. The process of malaria transmission involves complex transitions between mosquito vector and vertebrate host, involving multiple parasite stage transitions.

**FIGURE 1:**
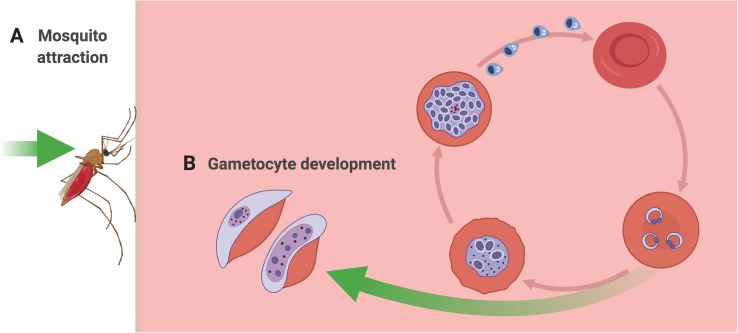
Metabolic windows into malaria transmission. **(A)** Mosquito attraction and feeding. Malaria-infected human hosts produce volatile metabolites (e.g. terpenes and aldehydes) through their skin and/or breath that may serve as attractants for the female *Anopheles* mosquito when she takes her blood meal. **(B)** Gametocyte development. Certain metabolites undergo dynamic fluctuations in the blood plasma during the course of a malaria infection. Some specific metabolite changes have been hypothesized to be detected by parasites *in vivo* and trigger increased conversion to gametocytes (e.g. amino acids and phospholipids).

Initiating a malaria infection in a human begins with the bite of a female *Anopheline* mosquito, which must locate and bite a human host in order to take a bloodmeal (Figure 1A). In doing so, the mosquito injects sporozoite stage parasites into the blood of the human host. Once inside the host, and following a period of development within the liver, the parasites begin to replicate asexually within red blood cells. At some point during the infection, a subset of parasites diverge down a path of sexual development, resulting in the formation of mature male and female gametocytes (Figure 1B). Importantly, mature gametocytes are the only stages that can be picked up by a mosquito and go on to successfully undergo fertilization and further development within the mosquito. Inside the mosquito, oocysts develop, and eventually burst, and sporozoites are released, migrating to the salivary gland, where they are now ready to be transmitted to the next human host during a subsequent bloodmeal. Adding to the complexity, recent studies suggest that metabolites present in the human host’s blood, skin, and breath affect the efficacy of each of the key transition points in the parasite’s life cycle, enhancing transmission. Specifically, volatile metabolites in the skin and breath of malaria-infected hosts have been shown to increase attractiveness to the mosquito vector (Figure 1A). And upstream of that biting event, metabolites in the host’s bloodstream appear to act as signals for parasites to increase their rate of developing transmissible gametocyte stages (Figure 1B). The following sections focus on how metabolites in the blood, skin and breath of malaria-infected hosts play key roles in malaria transmission through modulating (a) the attractiveness of hosts to mosquitoes and (b) the rate of gametocyte development within host blood.

### Metabolites Involved in Mosquito Attraction

It has been known for some time that carbon dioxide (CO_2_) and other volatile chemicals help mosquitoes locate their vertebrate hosts and that certain molecules released through the skin, sweat and/or breath of vertebrates are attractive to mosquitoes (Brouwer, 1960; Takken and Kline, 1989). Some of these compounds, such as 1-Octen-3-ol, are routinely manufactured into baited mosquito traps, for example. However, a key question still remaining regarding the transmission of malaria is – *do individuals with malaria have increased attractiveness to mosquitoes, as compared to uninfected people?* (Figure 1A).

Gas chromatography mass spectrometry (GCMS) has been employed in efforts to answer this question. A specific type of GCMS called head space gas chromatography mass spectrometry (HS-GCMS) is used to analyze volatile organic compounds (VOCs) in the air space coming off of the subject or sample in question. Human cross-sectional studies using this method in the context of malaria have focused on analyzing VOCs from the breath of malaria-infected individuals. This work, termed “breathprinting,” has demonstrated increased levels of multiple terpenes (α-pinene, limonene, and 3-carene), which are known mosquito-attractants (Nyasembe et al., 2012), in the breath of malaria-infected hosts (Kelly et al., 2015; Schaber et al., 2018) (Table 1). Additionally, controlled human malaria infection (CHMI) studies have also employed HS-GCMS to study the breath and skin odor composition in malaria-infected subjects (Berna et al., 2015; de Boer et al., 2017). In breath, four thioethers were found to increase in breath alongside increases in malaria parasitemia in blood: allyl methyl sulfide, 1-methylthio-propane, (*Z*)-1-methylthio-1-propene, and (*E*)-1-methylthio-1-propene (Berna et al., 2015). In skin, volatile compounds 2- and 3-methylbutanal, 3-hydroxy-2-butanone, and 6-methyl-5-hepten-2-one were all found to be present at higher levels in human skin odor during malaria infection, and also thought to attract mosquitoes (de Boer et al., 2017). In a further effort to identify VOCs derived from malaria parasites, an analysis of headspace from *in vitro Plasmodium* cultures was performed, and the headspace of the extracellular vesicles (EV) fraction also revealed an enrichment of a previously reported mosquito attractant aldehyde, hexanal (Correa et al., 2017) (Table 1).

**TABLE 1:** Metabolites associated with enhanced transmission of malaria.

**Role**	**Context**	**Method**	**Metabolite**	**Citation**
Increased mosquito attraction/feeding	mouse whole body headspace	GC	*Alkane*: tridecane	De Moraes et al., 2014
			*Fatty acids*: 3-methyl butanoic acid, 2-methyl butanoic acid, hexanoic acid	
	*In vitro Plasmodium* headspace	GC	*Terpenes*: pinene, limonene	Kelly et al., 2015
	*In vitro* HMBPP-stimulated RBC headspace	GC	*Aldehydes*: octanal, nonanal, decanal *Terpenes*: α- and β-pinene, limonene	Emami et al., 2017
			*Oxide:*CO_2_	
	human skin (foot) headspace	GC	*Aldehyde*: 3-methylbutanal	de Boer et al., 2017
			*Ketones*: 6-methyl-5-hepten-2-one, 2- and 3-hydroxy-2-butanone	
	*In vitro Plasmodium* headspace	GC	*Aldehyde*: hexanal	Correa et al., 2017
	human skin (foot) headspace	GC-EAG	*Aldehydes:* heptanal, octanal, nonanal	Robinson et al., 2018
	human breath	GC	*Terpenes:* α-pinene and 3-carene	Schaber et al., 2018
	human skin (foot) headspace	GC-EAG	*Aldehydes:* hexanal, nonanal	Stanczyk et al., 2019
			*Aromatic hydrocarbon*: toluene	
Increased gametocyte development	*In vitro Plasmodium*	LC	*α-amino acid:* homocysteine	Beri et al., 2017
	*in vitro Plasmodium*, human blood	LC	*Phospholipid:*lysophosphotidylcholine	Brancucci et al., 2017;
				Usui et al., 2019
	*In vitro Plasmodium*	LC	*Phospholipids:*polyunsaturated fatty acid-containing phospholipids	Tanaka et al., 2019

Taking this concept a step further, a subset of studies have combined HS-GCMS with functional sensory tests in order to identify specific components of the metabolome that are detected by a mosquito’s antenna. This approach, termed gas chromatography electroantennography (GC-EAG) involves the coupling of two approaches – chromatography-based compound analysis and an invertebrate sensory functional assessment. In short, as a compound comes off the column, it goes past a mosquito antenna. An olfactory nerve response occurs in the antenna, allowing for the determination of EAG-active metabolites. Example studies using GC-EAG include the analysis of foot odor of malaria-infected individuals, which revealed a set of aldehydes including heptanal, octanal, and nonanal produced at higher levels in malaria-infected individuals and shown to be sensed by mosquitoes (Robinson et al., 2018). Studies involving mammalian hosts including rodents have also used a combination of HS-GCMS and functional sensory tests and have demonstrated that 3-methyl butanoic acid, 2-methyl butanoic acid, hexanoic acid, and tridecane were all elevated at phases of mosquito attraction (De Moraes et al., 2014) (Table 1).

Olfactometers, devices used to test for host attractiveness to mosquitoes, have also been used to demonstrate that malaria-infected individuals are more attractive to mosquitoes. Of particular interest, this method has been used to demonstrate that humans carrying *Plasmodium* gametocytes have higher mosquito attractiveness than humans carrying asexual stages alone (Lacroix et al., 2005; Batista et al., 2014; Busula et al., 2017). Importantly this was seen in both *Plasmodium falciparum* and *P. vivax*. In addition, a similar approach was applied in a study of birds, which revealed that birds with chronic malaria are more attractive to mosquito vectors than those with acute malaria (Cornet et al., 2013).

While the production of specific VOCs seems to be linked to the presence of malaria parasites and particularly gametocytes within the mammalian hosts, the attraction by mosquitoes toward those hosts similarly appears to be affected by the infection status of the *mosquito*. Using olfactometers and neurophysiological assays, mosquitoes of the genus *Anopheles* were found to have a higher attractiveness toward human odors when they were infected with and carrying *Plasmodium* sporozoites than mosquitoes that were uninfected or were infected with those stages of the parasite that were not infectious yet (e.g. oocysts) (Cator et al., 2013; Smallegange et al., 2013). A recent study using GC-EAG to assess this question identified multiple compounds that infected mosquitoes responded to at a higher rate than uninfected mosquitoes, including some of the aldehydes identified in previous studies: hexanal and nonanal (Stanczyk et al., 2019) (Table 1). Further work combining olfactory tests with metabolomics approaches are likely to shed light on the full set of volatile chemicals that emanate from malaria-infected individuals, and can lead to broader characterization of compounds that increase host attractiveness to the mosquito vector.

### Metabolites Involved in Gametocyte Development

While the attraction of mosquitoes to human hosts is a major part of malaria transmission, another key aspect is the production of gametocytes, which need to be present in the bloodstream of the human host in order to successfully transmit the parasite to the mosquito vector. The process by which asexually reproducing malaria parasites switch to sexual development is called gametocytogenesis and appears to involve both genetic and environmental cues. Specific transcriptional cascades are turned on during the transition from asexual to sexual replication, and environmental changes in host physiology have been long thought to contribute to this process (Josling and Llinás, 2015). A combination of mass spectrometry-based metabolomics, metabolic labeling approaches, and functional assays have implicated a role for exogenous metabolites in triggering sexual development. The primary question here being *– what are the signals in host blood that trigger Plasmodium to undergo gametocytogenesis?* (Figure 1B).

Liquid chromatography mass spectrometry (LC-MS) has been utilized in a number of studies to identify metabolites within *in vitro* culture media that are taken up or produced by *Plasmodium* parasites. Studies have been done to investigate the metabolic needs of asexually replicating *Plasmodium* parasites *in vitro* (Olszewski et al., 2009). Additional studies have focused on metabolites in human blood that are altered during malaria infections (Basant et al., 2010; Pappa et al., 2015; Surowiec et al., 2015; Decuypere et al., 2016; Gardinassi et al., 2017; Uppal et al., 2017; Ghosh et al., 2018; Cordy et al., 2019). This body of work has demonstrated the utility of this tool for identifying a wide range of physiological changes that occur in vertebrate hosts during malaria infection.

In addition to these aforementioned studies which focus on asexual replication, a subset of investigations focus on how host metabolites affect disease transmission. For example, a combination of *in vitro* and *in vivo* studies has identified a role for homocysteine in sexual development. This α-amino acid has been shown to accumulate within *Plasmodium*-infected red blood cells *in vitro*, causing redox stress and triggering gametocytogenesis (Chaubey et al., 2014; Beri et al., 2017). Validating the role of this metabolite, homocysteine was also administered to mice with *P. berghei* and an increase in gametocytes was subsequently observed *in vivo* (Beri et al., 2017) (Table 1).

Another finding identified through *in vitro* studies paired with LCMS was the discovery that a decline in lysophosphotidylcholine species (lysoPC) in the media was associated with an increased rate of gametocytogenesis (Brancucci et al., 2017). This finding was also supported by LCMS and Flow Injection Analysis (FIA) data from human subjects, demonstrating that *Plasmodium* grown in serum from subjects with lower lysoPC resulted in higher rates of gametocyte development *in vitro* (Usui et al., 2019). Finally, another LCMS-based *in vitro* study examined the difference between human serum and AlbuMAX supplementation in media and found that higher levels of polyunsaturated fatty acid (PUFA)-containing phospholipids in the media were associated with higher rates of gametocyte development *in vitro* (Tanaka et al., 2019) (Table 1). Altogether, through the use of LCMS alongside *in vitro* and/or *in vivo* methodologies, multiple host metabolic signals (including α-amino acids and phospholipids) have been identified that appear to trigger gametocyte development, and thus could contribute to malaria transmission.

## Who Makes What? Complex Origins of Host Metabolites During Malaria Infection

Metabolites found in the blood, skin and breath of malaria-infected hosts do not all come from the host. The host, its infecting parasite and even its commensal microbes can all contribute to the metabolome during infection. A key question, therefore, about the metabolites identified in the aforementioned approaches is *– are these metabolites coming from the parasite or the host (or the host’s microbiome)?*

*Plasmodium* exhibits certain metabolic pathways that are not present in the vertebrate host, and chemicals of such pathways are thus known to be produced by the parasite. One such pathway is the 2-*C*-methyl-d-erythritol 4-phosphate (MEP) pathway, and one such chemical is (*E*)-4-hydroxy-3-methyl-but-2-enyl pyrophosphate (HMBPP), a precursor of the parasite’s MEP pathway. While HMBPP itself is not a mosquito attractant molecule, HMBPP plays a role in malaria transmission through a complex interaction between the parasite and the host’s red blood cells. HMBPP, which is produced by the parasite, has been shown to stimulate host red blood cells to increase their production of CO_2_, aldehydes and monoterpenes, thereby indirectly affecting mosquito attractiveness (Emami et al., 2017) (Table 1). The volatile metabolites that attract the mosquito are thus host-derived, but the signal that stimulated the metabolite production is of parasite origin.

Commensal microbes of vertebrate hosts also demonstrate complex interplays leading to the production of mosquito attractant molecules, as has been reviewed previously (Braks et al., 1999). For example, 2- and 3-methylbutanal and 3-hydroxy-2-butanone, mosquito attractants found in the skin odor of malaria-infected individuals (de Boer et al., 2017) (Table 1), had been shown previously to be produced by skin microbes (Verhulst et al., 2009). In this study, GCMS identified these metabolites in the headspace of skin-associated microbes which were grown *in vitro* (Verhulst et al., 2009). This work suggests that the host’s commensal skin microbes may be the source of several important mosquito-attractant compounds. Future work involving parallel investigations of skin microbiota and skin headspace VOCs during malaria infection may shed light on the significance of skin commensal microbes in the context of malaria transmission.

## Data Wrangling: Bioinformatics Approaches for Tackling the Malaria Infection Metabolome

Bioinformatics tools are critically important in the analysis of big data that arises from ‘omic technologies. Metabolomics, particularly untargeted metabolomics, can yield a large amount of data on many unique metabolic features which must be sifted out and sorted through in order to determine meaning. A key question here is *– how to find signal within the noise in a metabolomics data set?*

A combination of targeted and untargeted approaches has been used to explore the question of host metabolites in the context of malaria. The strength of untargeted analyses is that these approaches enable the analysis of an extremely large set of metabolic features, allowing for unbiased assessments without setting an *a priori* hypotheses about what may be found. Unsupervised statistical analyses can be applied to these big datasets, such as principal components analysis (PCA) and hierarchical clustering analysis (HCA), and metabolic features that are highly associated with the phenomenon of interest can be identified based on their chemical features (e.g. mass:charge ratio). From that point, computational annotation approaches can be applied to give putative identities, and pathway analysis can be performed to test for significantly perturbed metabolic pathways (Uppal et al., 2016). These approaches can be helpful in the context of exploring new and under-researched questions in the field. Ultimately, however, these approaches should be combined with targeted methods and reference compounds to confirm identities of any putative metabolites (Uppal et al., 2017; Cordy et al., 2019).

Biomarker discovery is another key area in which bioinformatic approaches are applied to metabolomics data. While often used in the context of disease pathogenesis, metabolites could also potentially be identified as biomarkers to indicate malaria transmissibility. To identify metabolite biomarkers of malaria transmission, approaches such as receiver operating curve (ROC) analysis, can be used. More advanced approaches that incorporate multivariate statistics may also be used to help identify robust biomarkers. Importantly these studies require validation in multiple cohorts, and the use of independent datasets on which to build and subsequently apply any given model. Such bioinformatics approaches are likely to lead to the discovery of new biomarkers of transmissibility, that could be highly relevant during the era of malaria eradication.

Potential biomarkers could include those metabolites which have already been found in multiple studies to be associated with enhanced transmissibility. Biomarkers for increased mosquito attraction may include elevated levels of certain terpenes (e.g. pinene) (Kelly et al., 2015; Emami et al., 2017; Schaber et al., 2018) and/or aldehydes (e.g. hexanal, octanal, nonanal) (Correa et al., 2017; Emami et al., 2017; Robinson et al., 2018; Stanczyk et al., 2019) in the headspace of malaria-infected individuals. Biomarkers for increased gametocytogenesis may include altered levels of certain phospholipids (e.g. lysoPC, PUFA-containing phospholipids) (Brancucci et al., 2017; Tanaka et al., 2019; Usui et al., 2019) in the blood of malaria-infected hosts. The utility of such biomarkers is that there may be potential to generate diagnostic tests, particularly non-invasive ones, that detect relevant biomarkers for malaria infection and/or transmissibility. Further, as demonstrated by a recent study of canines identifying malaria-infected individuals through the smell of their socks alone (Guest et al., 2019), it is apparent that volatile chemicals can be detected in a wide variety of ways, and the successful detection of these types of biomarkers may not require expensive equipment.

Finally, in trying to understand the source of these metabolites, data integration across multiple ‘omic analyses derived from the same experiment can be helpful toward determining possible sources of metabolites within the complex milieu of the host-parasite-microbial system (Salinas et al., 2014). Data integration techniques can aid in sifting through big datasets to gauge which metabolites are coming from the parasite and which are coming from host (and possibly which from its commensal microbiota). Combining transcriptional data from host, parasite, and microbiota with metabolomics data from the complex host-parasite-microbe system may help to elucidate the ways in which these different components may contribute to the resulting metabolites. Metabolic flux models are particularly helpful for these such joint analyses, as they can combine transcriptional data (which is annotated and thus of known origin) with metabolomics data (from unknown origin). Putting these data together can help to illustrate the predicted activity of various metabolic pathways of either the host or parasite (Chiappino-Pepe et al., 2017; Tang et al., 2018).

## Conclusion

*Plasmodium* is a highly complex pathogen which undergoes multiple stage transitions during its passage between its invertebrate and vertebrate hosts. And despite (or more likely, due to) this complexity, transmission of the pathogen results in over 200 million cases of malaria per year (World Health Organization [WHO], 2018). Host, parasite and microbial-derived metabolites, through their impacts on mosquito attractiveness and gametocytogenesis, appear to play a critical role in ensuring the successful transmission of this devastating parasitic disease. While several recent studies have greatly added to our knowledge in this area, there are still many aspects of malaria transmission biology that are not well understood. Further mining of the host metabolome may help to improve our understanding of the complexities that exist in the malaria host-pathogen system that enhance or hinder transmission.

## Author Contributions

RJ conceived and wrote the manuscript.

## Conflict of Interest

The authors declare that the research was conducted in the absence of any commercial or financial relationships that could be construed as a potential conflict of interest.
